# Optical super-high magnification dermoscopy *versus* standard dermoscopy in basal cell carcinoma^☆^

**DOI:** 10.1016/j.abd.2025.501152

**Published:** 2025-07-03

**Authors:** Izadora Moreira do Amaral, Elisa Scandiuzzi Maciel, Daniela Surjan Milheti, Camila Arai Seque, Milvia Maria Simões e Silva Enokihara, Sérgio Henrique Hirata

**Affiliations:** a Department of Dermatology, Escola Paulista de Medicina, Universidade Federal de São Paulo, São Paulo, SP, Brazil; b Department of Pathology, Escola Paulista de Medicina, Universidade Federal de São Paulo, São Paulo, SP, Brazil

*Dear Editor,*

The recent emergence of super high dermoscopy (SHD) allows magnifications of up to 400 times, which brings new perspectives to the interpretation of dermoscopic images. This technology is available with non-polarized light and the images are obtained using the Fotofinder Medicam 1000 device (Fotofinder System, Bad Birnbach, Germany) replacing the conventional terminal lens with the super high dermoscopy (SHD) lens.

To demonstrate the potential of using SHD, this report describes the case of a 78-year-old female patient with Fitzpatrick skin phototype III, with a brownish papule in the left preauricular region with progressive growth. The patient was photographed using conventional dermoscopy and super high dermoscopy with ultrasound gel immersion (Figure 1, Figure 2, Figure 3). The patient was subsequently referred for tumor excision and the material was sent for histopathological examination, with a report compatible with pigmented solid basal cell carcinoma with an adenoid component.

**Figure 1:**
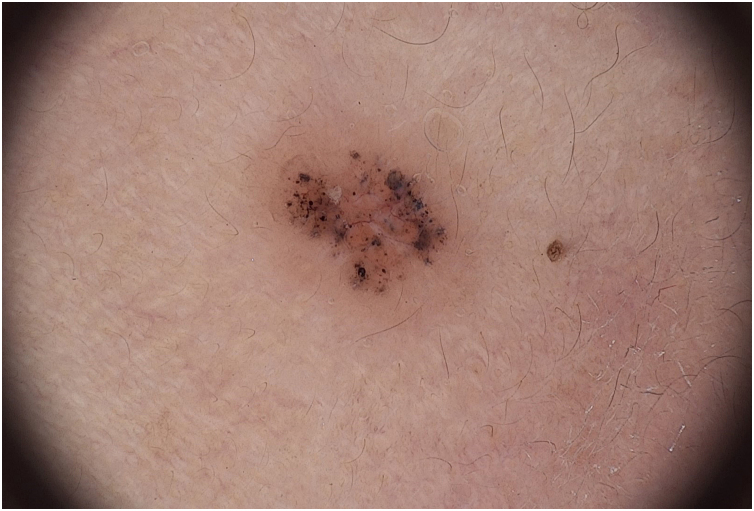
Digital dermoscopy with x20 magnification. Fotofinder System, Bad Birnbach, Germany.

**Figure 2:**
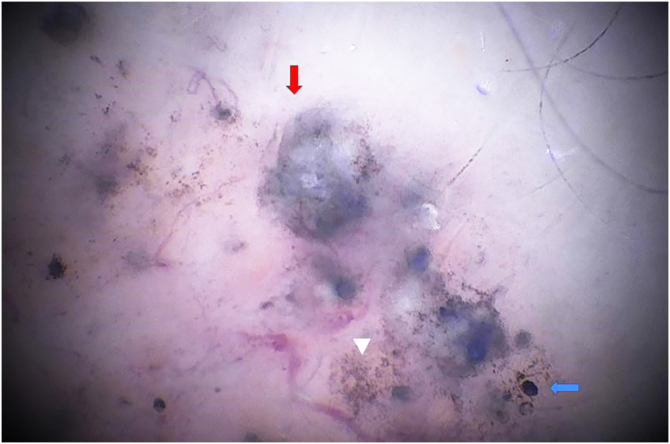
SHD with x180 magnification. The image shows blue ovoid nests at higher magnification (red arrow), rounded blue globules (blue arrow) and irregular pigmented structures (triangle).

**Figure 3:**
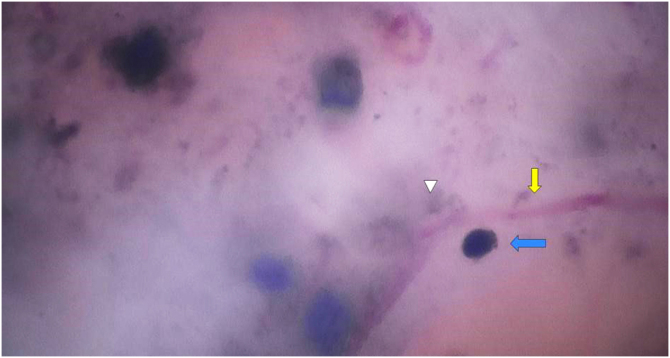
SHD of the same region with x400 magnification. Telangiectasias can be seen at higher magnification, with focus (yellow arrow), surrounded by irregular pigmented structures (triangle) and rounded blue globules (blue arrow).

SHD allows the visualization of structures that are not perceptible through conventional dermoscopy.^1^ In the literature, there are reports on the use of SHD in the identification and differentiation between melanomas and atypical nevi in ​​melanocytic lesions,^2^ as well as in the differential diagnosis between benign facial lesions and lentigo maligna.^3^ Regarding basal cell carcinomas, irregularly pigmented structures, corresponding to melanocyte deposits containing melanin,^4^ linear vessels with peripheral dots and globules,^5^ vessels with a pattern similar to oak leaves,^6^ and hairpin vessels,^4^ have already been described exclusively through SHD.

The case described herein illustrates the easier identification of dermoscopic structures observed by SHD when compared to conventional dermoscopy. In Fig. 1, conventional dermoscopic examination (×20 magnification) allows the visualization of structures that less experienced examiners may mistake for globules of melanocytic lesions. Figure 2, Figure 3 show the same structures observed at SHD (x180 and x400 magnification). It is clear that these are bluish-gray globules, structures that are characteristic of basal cell carcinomas. The morphological characteristic of telangiectasias is also more easily observed at SHD, with focus and showing the characteristic peripheral dots previously described for SHD.^5^ Fig. 4, for comparison purposes, shows structures in an intradermal nevus with a tendency to an annular arrangement (globule), corresponding to nests formed by the union of nevus cells.

**Figure 4:**
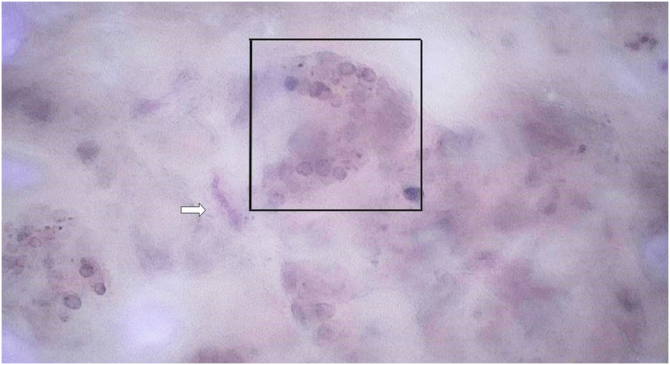
SHD at x400 magnification demonstrates a globule of an intradermal melanocytic nevus. Telangiectasias without focus (white arrow) can be observed.

The nomenclature and description of the different structures observed at SHD are not yet standardized and the use of this technique is still in the experimental phase. It is not a substitute for conventional dermoscopy, but rather a new tool capable of aiding in diagnoses through additional information.

## Financial support

None declared.

## Authors' contributions

Izadora Moreira do Amaral: Collection, analysis and interpretation of data; intellectual participation in the propaedeutic and/or therapeutic conduct of the studied cases; drafting and editing of the manuscript or critical review of important intellectual content; critical review of the literature; approval of the final version of the manuscript.

Elisa Scandiuzzi Maciel: Collection, analysis and interpretation of data; intellectual participation in the propaedeutic and/or therapeutic conduct of the studied cases; approval of the final version of the manuscript.

Daniela Surjan Milheti: Collection, analysis and interpretation of data; intellectual participation in the propaedeutic and/or therapeutic conduct of the studied cases; approval of the final version of the manuscript.

Camila Arai Seque: Collection, analysis and interpretation of data; intellectual participation in the propaedeutic and/or therapeutic conduct of the studied cases; approval of the final version of the manuscript.

Milvia Maria Simões e Silva Enokihara: Collection, analysis and interpretation of data; drafting and editing of the manuscript or critical review of important intellectual content; effective participation in research orientation; intellectual participation in the propaedeutic and/or therapeutic conduct of the studied cases; approval of the final version of the manuscript.

Sérgio Henrique Hirata: Collection, analysis and interpretation of data; drafting and editing of the manuscript or critical review of important intellectual content; effective participation in research orientation; intellectual participation in the propaedeutic and/or therapeutic conduct of the studied cases; approval of the final version of the manuscript.

## Conflicts of interest

None declared.
